# Supplementary material for: Optimal minimal residual disease threshold in pediatric acute myeloid leukemia: A retrospective cohort study based on the TARGET database
Source: PLoS Med. 2026 May 8;23(5):e1005088. doi: 10.1371/journal.pmed.1005088 (PMC13155632; doi:10.1371/journal.pmed.1005088)
Supplement: S1 Code — (ZIP) [file pmed.1005088.s002.zip › S2 code/PROJ8_22_tbl1/PROJ8_22_tbl1.htm]

## Kaplan Meier Survival Curve

Outcome: First Event
Time: EFS(years)
Landmark analysis for time segment: EFS(years) <= 5
Log rank test: implements the G-rho family of Harrington and Fleming (1982), with weights on each death of S(t)^rho, where S is the Kaplan-Meier estimate of survival. With rho = 0 this is the log-rank or Mantel-Haenszel test.

|  |  |  |  |
| --- | --- | --- | --- |
|  | N | Observed | Expected |
| factor(X23.CS2)=0 | 906 | 378 | 494.9954 |
| factor(X23.CS2)=1 | 18 | 15 | 8.3232 |
| factor(X23.CS2)=2 | 281 | 210 | 99.6814 |

Chisq=
156.7435
on
2
degree of freedom, p=
<0.0001
Survival table

|  |  |  |  |  |  |  |  |
| --- | --- | --- | --- | --- | --- | --- | --- |
|  | EFS(years) | N.Risk | N.Event | N.Censor | Survival | 95%CI Low | 95%CI Upp |
| factor(X23.CS2)=0 | 0.16 | 906 | 1 | 0 | 0.9989 | 0.9967 | 1.0000 |
| factor(X23.CS2)=0 | 0.17 | 905 | 3 | 0 | 0.9956 | 0.9913 | 0.9999 |
| factor(X23.CS2)=0 | 0.18 | 902 | 2 | 0 | 0.9934 | 0.9881 | 0.9987 |
| factor(X23.CS2)=0 | 0.19 | 900 | 1 | 1 | 0.9923 | 0.9866 | 0.9980 |
| factor(X23.CS2)=0 | 0.2 | 898 | 4 | 0 | 0.9879 | 0.9807 | 0.9950 |
| factor(X23.CS2)=0 | 0.21 | 894 | 4 | 0 | 0.9834 | 0.9752 | 0.9918 |
| factor(X23.CS2)=0 | 0.22 | 890 | 1 | 0 | 0.9823 | 0.9738 | 0.9909 |
| factor(X23.CS2)=0 | 0.23 | 889 | 2 | 1 | 0.9801 | 0.9711 | 0.9893 |
| factor(X23.CS2)=0 | 0.24 | 886 | 1 | 0 | 0.9790 | 0.9697 | 0.9884 |
| factor(X23.CS2)=0 | 0.26 | 885 | 4 | 0 | 0.9746 | 0.9644 | 0.9849 |
| factor(X23.CS2)=0 | 0.27 | 881 | 2 | 1 | 0.9724 | 0.9618 | 0.9831 |
| factor(X23.CS2)=0 | 0.28 | 878 | 4 | 0 | 0.9679 | 0.9565 | 0.9795 |
| factor(X23.CS2)=0 | 0.29 | 874 | 3 | 0 | 0.9646 | 0.9527 | 0.9767 |
| factor(X23.CS2)=0 | 0.31 | 871 | 1 | 0 | 0.9635 | 0.9514 | 0.9758 |
| factor(X23.CS2)=0 | 0.32 | 870 | 2 | 0 | 0.9613 | 0.9488 | 0.9740 |
| factor(X23.CS2)=0 | 0.33 | 868 | 2 | 0 | 0.9591 | 0.9463 | 0.9721 |
| factor(X23.CS2)=0 | 0.37 | 866 | 2 | 0 | 0.9569 | 0.9437 | 0.9702 |
| factor(X23.CS2)=0 | 0.38 | 864 | 2 | 0 | 0.9547 | 0.9412 | 0.9683 |
| factor(X23.CS2)=0 | 0.4 | 862 | 4 | 0 | 0.9502 | 0.9362 | 0.9645 |
| factor(X23.CS2)=0 | 0.41 | 858 | 2 | 0 | 0.9480 | 0.9336 | 0.9626 |
| factor(X23.CS2)=0 | 0.42 | 856 | 2 | 0 | 0.9458 | 0.9311 | 0.9607 |
| factor(X23.CS2)=0 | 0.44 | 854 | 5 | 0 | 0.9403 | 0.9249 | 0.9558 |
| factor(X23.CS2)=0 | 0.45 | 849 | 2 | 0 | 0.9380 | 0.9225 | 0.9539 |
| factor(X23.CS2)=0 | 0.46 | 847 | 2 | 0 | 0.9358 | 0.9200 | 0.9519 |
| factor(X23.CS2)=0 | 0.47 | 845 | 3 | 0 | 0.9325 | 0.9163 | 0.9490 |
| factor(X23.CS2)=0 | 0.5 | 842 | 3 | 0 | 0.9292 | 0.9126 | 0.9461 |
| factor(X23.CS2)=0 | 0.51 | 839 | 1 | 0 | 0.9281 | 0.9114 | 0.9451 |
| factor(X23.CS2)=0 | 0.52 | 838 | 3 | 0 | 0.9248 | 0.9077 | 0.9421 |
| factor(X23.CS2)=0 | 0.53 | 835 | 5 | 0 | 0.9192 | 0.9016 | 0.9372 |
| factor(X23.CS2)=0 | 0.54 | 830 | 2 | 0 | 0.9170 | 0.8992 | 0.9352 |
| factor(X23.CS2)=0 | 0.55 | 828 | 3 | 0 | 0.9137 | 0.8955 | 0.9322 |
| factor(X23.CS2)=0 | 0.56 | 825 | 6 | 0 | 0.9070 | 0.8883 | 0.9262 |
| factor(X23.CS2)=0 | 0.57 | 819 | 4 | 0 | 0.9026 | 0.8835 | 0.9221 |
| factor(X23.CS2)=0 | 0.58 | 815 | 4 | 0 | 0.8982 | 0.8787 | 0.9181 |
| factor(X23.CS2)=0 | 0.59 | 811 | 4 | 0 | 0.8937 | 0.8739 | 0.9141 |
| factor(X23.CS2)=0 | 0.6 | 807 | 4 | 0 | 0.8893 | 0.8691 | 0.9100 |
| factor(X23.CS2)=0 | 0.61 | 803 | 1 | 0 | 0.8882 | 0.8679 | 0.9090 |
| factor(X23.CS2)=0 | 0.62 | 802 | 2 | 0 | 0.8860 | 0.8655 | 0.9070 |
| factor(X23.CS2)=0 | 0.63 | 800 | 1 | 0 | 0.8849 | 0.8643 | 0.9059 |
| factor(X23.CS2)=0 | 0.64 | 799 | 4 | 0 | 0.8805 | 0.8595 | 0.9019 |
| factor(X23.CS2)=0 | 0.65 | 795 | 4 | 0 | 0.8760 | 0.8548 | 0.8978 |
| factor(X23.CS2)=0 | 0.66 | 791 | 1 | 0 | 0.8749 | 0.8536 | 0.8968 |
| factor(X23.CS2)=0 | 0.67 | 790 | 3 | 0 | 0.8716 | 0.8500 | 0.8937 |
| factor(X23.CS2)=0 | 0.68 | 787 | 5 | 0 | 0.8661 | 0.8441 | 0.8886 |
| factor(X23.CS2)=0 | 0.69 | 782 | 4 | 0 | 0.8616 | 0.8394 | 0.8844 |
| factor(X23.CS2)=0 | 0.7 | 778 | 3 | 0 | 0.8583 | 0.8359 | 0.8814 |
| factor(X23.CS2)=0 | 0.71 | 775 | 3 | 0 | 0.8550 | 0.8323 | 0.8783 |
| factor(X23.CS2)=0 | 0.72 | 772 | 5 | 0 | 0.8494 | 0.8264 | 0.8731 |
| factor(X23.CS2)=0 | 0.73 | 767 | 4 | 0 | 0.8450 | 0.8217 | 0.8689 |
| factor(X23.CS2)=0 | 0.74 | 763 | 4 | 0 | 0.8406 | 0.8170 | 0.8648 |
| factor(X23.CS2)=0 | 0.75 | 759 | 2 | 0 | 0.8384 | 0.8147 | 0.8627 |
| factor(X23.CS2)=0 | 0.76 | 757 | 8 | 0 | 0.8295 | 0.8053 | 0.8544 |
| factor(X23.CS2)=0 | 0.77 | 749 | 1 | 0 | 0.8284 | 0.8042 | 0.8534 |
| factor(X23.CS2)=0 | 0.78 | 748 | 2 | 0 | 0.8262 | 0.8018 | 0.8513 |
| factor(X23.CS2)=0 | 0.79 | 746 | 6 | 0 | 0.8195 | 0.7948 | 0.8450 |
| factor(X23.CS2)=0 | 0.8 | 740 | 4 | 0 | 0.8151 | 0.7902 | 0.8408 |
| factor(X23.CS2)=0 | 0.81 | 736 | 5 | 0 | 0.8096 | 0.7844 | 0.8356 |
| factor(X23.CS2)=0 | 0.82 | 731 | 6 | 0 | 0.8029 | 0.7774 | 0.8293 |
| factor(X23.CS2)=0 | 0.83 | 725 | 4 | 0 | 0.7985 | 0.7728 | 0.8251 |
| factor(X23.CS2)=0 | 0.84 | 721 | 5 | 0 | 0.7930 | 0.7670 | 0.8198 |
| factor(X23.CS2)=0 | 0.85 | 716 | 1 | 0 | 0.7919 | 0.7658 | 0.8188 |
| factor(X23.CS2)=0 | 0.86 | 715 | 2 | 0 | 0.7896 | 0.7635 | 0.8167 |
| factor(X23.CS2)=0 | 0.87 | 713 | 1 | 0 | 0.7885 | 0.7623 | 0.8156 |
| factor(X23.CS2)=0 | 0.88 | 712 | 7 | 0 | 0.7808 | 0.7543 | 0.8082 |
| factor(X23.CS2)=0 | 0.89 | 705 | 2 | 0 | 0.7786 | 0.7520 | 0.8061 |
| factor(X23.CS2)=0 | 0.9 | 703 | 3 | 0 | 0.7752 | 0.7485 | 0.8029 |
| factor(X23.CS2)=0 | 0.91 | 700 | 2 | 0 | 0.7730 | 0.7462 | 0.8008 |
| factor(X23.CS2)=0 | 0.92 | 698 | 4 | 0 | 0.7686 | 0.7416 | 0.7966 |
| factor(X23.CS2)=0 | 0.93 | 694 | 8 | 0 | 0.7597 | 0.7324 | 0.7881 |
| factor(X23.CS2)=0 | 0.94 | 686 | 2 | 0 | 0.7575 | 0.7301 | 0.7860 |
| factor(X23.CS2)=0 | 0.95 | 684 | 1 | 0 | 0.7564 | 0.7289 | 0.7849 |
| factor(X23.CS2)=0 | 0.96 | 683 | 2 | 1 | 0.7542 | 0.7266 | 0.7828 |
| factor(X23.CS2)=0 | 0.97 | 680 | 1 | 0 | 0.7531 | 0.7255 | 0.7817 |
| factor(X23.CS2)=0 | 0.99 | 679 | 2 | 1 | 0.7509 | 0.7232 | 0.7796 |
| factor(X23.CS2)=0 | 1 | 676 | 4 | 0 | 0.7464 | 0.7186 | 0.7754 |
| factor(X23.CS2)=0 | 1.01 | 672 | 3 | 0 | 0.7431 | 0.7151 | 0.7721 |
| factor(X23.CS2)=0 | 1.02 | 669 | 3 | 1 | 0.7398 | 0.7117 | 0.7689 |
| factor(X23.CS2)=0 | 1.03 | 665 | 3 | 0 | 0.7364 | 0.7082 | 0.7657 |
| factor(X23.CS2)=0 | 1.05 | 662 | 6 | 0 | 0.7298 | 0.7014 | 0.7593 |
| factor(X23.CS2)=0 | 1.06 | 656 | 2 | 1 | 0.7275 | 0.6991 | 0.7572 |
| factor(X23.CS2)=0 | 1.07 | 653 | 4 | 0 | 0.7231 | 0.6945 | 0.7529 |
| factor(X23.CS2)=0 | 1.08 | 649 | 3 | 0 | 0.7197 | 0.6910 | 0.7496 |
| factor(X23.CS2)=0 | 1.09 | 646 | 1 | 0 | 0.7186 | 0.6899 | 0.7486 |
| factor(X23.CS2)=0 | 1.1 | 645 | 2 | 0 | 0.7164 | 0.6876 | 0.7464 |
| factor(X23.CS2)=0 | 1.11 | 643 | 0 | 2 | 0.7164 | 0.6876 | 0.7464 |
| factor(X23.CS2)=0 | 1.12 | 641 | 3 | 1 | 0.7130 | 0.6841 | 0.7432 |
| factor(X23.CS2)=0 | 1.13 | 637 | 4 | 0 | 0.7086 | 0.6795 | 0.7388 |
| factor(X23.CS2)=0 | 1.14 | 633 | 2 | 1 | 0.7063 | 0.6772 | 0.7367 |
| factor(X23.CS2)=0 | 1.15 | 630 | 1 | 1 | 0.7052 | 0.6761 | 0.7356 |
| factor(X23.CS2)=0 | 1.16 | 628 | 1 | 0 | 0.7041 | 0.6749 | 0.7345 |
| factor(X23.CS2)=0 | 1.17 | 627 | 1 | 0 | 0.7030 | 0.6738 | 0.7334 |
| factor(X23.CS2)=0 | 1.18 | 626 | 1 | 0 | 0.7018 | 0.6726 | 0.7323 |
| factor(X23.CS2)=0 | 1.19 | 625 | 2 | 0 | 0.6996 | 0.6703 | 0.7302 |
| factor(X23.CS2)=0 | 1.2 | 623 | 3 | 0 | 0.6962 | 0.6668 | 0.7269 |
| factor(X23.CS2)=0 | 1.21 | 620 | 1 | 1 | 0.6951 | 0.6657 | 0.7258 |
| factor(X23.CS2)=0 | 1.24 | 618 | 1 | 0 | 0.6940 | 0.6645 | 0.7247 |
| factor(X23.CS2)=0 | 1.25 | 617 | 0 | 1 | 0.6940 | 0.6645 | 0.7247 |
| factor(X23.CS2)=0 | 1.26 | 616 | 1 | 0 | 0.6928 | 0.6634 | 0.7236 |
| factor(X23.CS2)=0 | 1.27 | 615 | 4 | 0 | 0.6883 | 0.6587 | 0.7193 |
| factor(X23.CS2)=0 | 1.28 | 611 | 2 | 1 | 0.6861 | 0.6564 | 0.7171 |
| factor(X23.CS2)=0 | 1.3 | 608 | 3 | 0 | 0.6827 | 0.6530 | 0.7138 |
| factor(X23.CS2)=0 | 1.32 | 605 | 1 | 0 | 0.6816 | 0.6518 | 0.7127 |
| factor(X23.CS2)=0 | 1.34 | 604 | 3 | 1 | 0.6782 | 0.6483 | 0.7094 |
| factor(X23.CS2)=0 | 1.35 | 600 | 1 | 0 | 0.6771 | 0.6472 | 0.7083 |
| factor(X23.CS2)=0 | 1.36 | 599 | 2 | 0 | 0.6748 | 0.6449 | 0.7061 |
| factor(X23.CS2)=0 | 1.37 | 597 | 2 | 1 | 0.6725 | 0.6426 | 0.7039 |
| factor(X23.CS2)=0 | 1.38 | 594 | 2 | 0 | 0.6703 | 0.6402 | 0.7017 |
| factor(X23.CS2)=0 | 1.39 | 592 | 2 | 0 | 0.6680 | 0.6379 | 0.6995 |
| factor(X23.CS2)=0 | 1.4 | 590 | 1 | 0 | 0.6669 | 0.6368 | 0.6984 |
| factor(X23.CS2)=0 | 1.41 | 589 | 1 | 0 | 0.6657 | 0.6356 | 0.6973 |
| factor(X23.CS2)=0 | 1.42 | 588 | 4 | 0 | 0.6612 | 0.6310 | 0.6929 |
| factor(X23.CS2)=0 | 1.43 | 584 | 4 | 0 | 0.6567 | 0.6264 | 0.6885 |
| factor(X23.CS2)=0 | 1.45 | 580 | 1 | 0 | 0.6555 | 0.6252 | 0.6874 |
| factor(X23.CS2)=0 | 1.48 | 579 | 2 | 0 | 0.6533 | 0.6229 | 0.6852 |
| factor(X23.CS2)=0 | 1.49 | 577 | 3 | 0 | 0.6499 | 0.6194 | 0.6818 |
| factor(X23.CS2)=0 | 1.51 | 574 | 1 | 0 | 0.6488 | 0.6183 | 0.6807 |
| factor(X23.CS2)=0 | 1.53 | 573 | 1 | 0 | 0.6476 | 0.6171 | 0.6796 |
| factor(X23.CS2)=0 | 1.54 | 572 | 1 | 0 | 0.6465 | 0.6160 | 0.6785 |
| factor(X23.CS2)=0 | 1.55 | 571 | 1 | 0 | 0.6454 | 0.6148 | 0.6774 |
| factor(X23.CS2)=0 | 1.56 | 570 | 1 | 0 | 0.6442 | 0.6137 | 0.6763 |
| factor(X23.CS2)=0 | 1.58 | 569 | 4 | 0 | 0.6397 | 0.6090 | 0.6719 |
| factor(X23.CS2)=0 | 1.6 | 565 | 2 | 0 | 0.6374 | 0.6067 | 0.6697 |
| factor(X23.CS2)=0 | 1.61 | 563 | 0 | 1 | 0.6374 | 0.6067 | 0.6697 |
| factor(X23.CS2)=0 | 1.62 | 562 | 2 | 0 | 0.6352 | 0.6044 | 0.6675 |
| factor(X23.CS2)=0 | 1.64 | 560 | 1 | 0 | 0.6340 | 0.6033 | 0.6663 |
| factor(X23.CS2)=0 | 1.65 | 559 | 2 | 0 | 0.6318 | 0.6010 | 0.6641 |
| factor(X23.CS2)=0 | 1.66 | 557 | 1 | 0 | 0.6306 | 0.5998 | 0.6630 |
| factor(X23.CS2)=0 | 1.67 | 556 | 1 | 0 | 0.6295 | 0.5987 | 0.6619 |
| factor(X23.CS2)=0 | 1.71 | 555 | 0 | 1 | 0.6295 | 0.5987 | 0.6619 |
| factor(X23.CS2)=0 | 1.72 | 554 | 2 | 0 | 0.6272 | 0.5964 | 0.6597 |
| factor(X23.CS2)=0 | 1.75 | 552 | 0 | 1 | 0.6272 | 0.5964 | 0.6597 |
| factor(X23.CS2)=0 | 1.77 | 551 | 1 | 0 | 0.6261 | 0.5952 | 0.6586 |
| factor(X23.CS2)=0 | 1.78 | 550 | 1 | 0 | 0.6249 | 0.5940 | 0.6574 |
| factor(X23.CS2)=0 | 1.81 | 549 | 1 | 0 | 0.6238 | 0.5929 | 0.6563 |
| factor(X23.CS2)=0 | 1.83 | 548 | 0 | 1 | 0.6238 | 0.5929 | 0.6563 |
| factor(X23.CS2)=0 | 1.87 | 547 | 1 | 1 | 0.6227 | 0.5917 | 0.6552 |
| factor(X23.CS2)=0 | 1.89 | 545 | 2 | 0 | 0.6204 | 0.5894 | 0.6530 |
| factor(X23.CS2)=0 | 1.9 | 543 | 2 | 0 | 0.6181 | 0.5871 | 0.6507 |
| factor(X23.CS2)=0 | 1.92 | 541 | 1 | 1 | 0.6170 | 0.5859 | 0.6496 |
| factor(X23.CS2)=0 | 1.94 | 539 | 1 | 2 | 0.6158 | 0.5848 | 0.6485 |
| factor(X23.CS2)=0 | 1.96 | 536 | 0 | 1 | 0.6158 | 0.5848 | 0.6485 |
| factor(X23.CS2)=0 | 1.98 | 535 | 0 | 1 | 0.6158 | 0.5848 | 0.6485 |
| factor(X23.CS2)=0 | 1.99 | 534 | 1 | 0 | 0.6147 | 0.5836 | 0.6474 |
| factor(X23.CS2)=0 | 2.03 | 533 | 1 | 0 | 0.6135 | 0.5824 | 0.6462 |
| factor(X23.CS2)=0 | 2.05 | 532 | 1 | 0 | 0.6123 | 0.5813 | 0.6451 |
| factor(X23.CS2)=0 | 2.07 | 531 | 1 | 0 | 0.6112 | 0.5801 | 0.6440 |
| factor(X23.CS2)=0 | 2.08 | 530 | 1 | 0 | 0.6100 | 0.5789 | 0.6428 |
| factor(X23.CS2)=0 | 2.09 | 529 | 1 | 0 | 0.6089 | 0.5777 | 0.6417 |
| factor(X23.CS2)=0 | 2.1 | 528 | 0 | 1 | 0.6089 | 0.5777 | 0.6417 |
| factor(X23.CS2)=0 | 2.11 | 527 | 1 | 0 | 0.6077 | 0.5766 | 0.6406 |
| factor(X23.CS2)=0 | 2.13 | 526 | 1 | 0 | 0.6066 | 0.5754 | 0.6394 |
| factor(X23.CS2)=0 | 2.14 | 525 | 1 | 0 | 0.6054 | 0.5742 | 0.6383 |
| factor(X23.CS2)=0 | 2.15 | 524 | 1 | 0 | 0.6043 | 0.5731 | 0.6372 |
| factor(X23.CS2)=0 | 2.18 | 523 | 0 | 1 | 0.6043 | 0.5731 | 0.6372 |
| factor(X23.CS2)=0 | 2.2 | 522 | 1 | 0 | 0.6031 | 0.5719 | 0.6360 |
| factor(X23.CS2)=0 | 2.22 | 521 | 2 | 1 | 0.6008 | 0.5695 | 0.6338 |
| factor(X23.CS2)=0 | 2.23 | 518 | 1 | 1 | 0.5996 | 0.5684 | 0.6326 |
| factor(X23.CS2)=0 | 2.27 | 516 | 0 | 2 | 0.5996 | 0.5684 | 0.6326 |
| factor(X23.CS2)=0 | 2.3 | 514 | 1 | 0 | 0.5985 | 0.5672 | 0.6315 |
| factor(X23.CS2)=0 | 2.31 | 513 | 1 | 0 | 0.5973 | 0.5660 | 0.6303 |
| factor(X23.CS2)=0 | 2.32 | 512 | 0 | 1 | 0.5973 | 0.5660 | 0.6303 |
| factor(X23.CS2)=0 | 2.34 | 511 | 1 | 0 | 0.5961 | 0.5648 | 0.6292 |
| factor(X23.CS2)=0 | 2.36 | 510 | 1 | 0 | 0.5950 | 0.5636 | 0.6280 |
| factor(X23.CS2)=0 | 2.37 | 509 | 1 | 1 | 0.5938 | 0.5624 | 0.6269 |
| factor(X23.CS2)=0 | 2.39 | 507 | 0 | 1 | 0.5938 | 0.5624 | 0.6269 |
| factor(X23.CS2)=0 | 2.4 | 506 | 0 | 1 | 0.5938 | 0.5624 | 0.6269 |
| factor(X23.CS2)=0 | 2.41 | 505 | 0 | 1 | 0.5938 | 0.5624 | 0.6269 |
| factor(X23.CS2)=0 | 2.42 | 504 | 1 | 0 | 0.5926 | 0.5612 | 0.6257 |
| factor(X23.CS2)=0 | 2.43 | 503 | 0 | 1 | 0.5926 | 0.5612 | 0.6257 |
| factor(X23.CS2)=0 | 2.44 | 502 | 0 | 2 | 0.5926 | 0.5612 | 0.6257 |
| factor(X23.CS2)=0 | 2.46 | 500 | 0 | 1 | 0.5926 | 0.5612 | 0.6257 |
| factor(X23.CS2)=0 | 2.47 | 499 | 0 | 1 | 0.5926 | 0.5612 | 0.6257 |
| factor(X23.CS2)=0 | 2.48 | 498 | 1 | 0 | 0.5914 | 0.5600 | 0.6246 |
| factor(X23.CS2)=0 | 2.49 | 497 | 0 | 1 | 0.5914 | 0.5600 | 0.6246 |
| factor(X23.CS2)=0 | 2.5 | 496 | 0 | 4 | 0.5914 | 0.5600 | 0.6246 |
| factor(X23.CS2)=0 | 2.51 | 492 | 1 | 1 | 0.5902 | 0.5588 | 0.6234 |
| factor(X23.CS2)=0 | 2.52 | 490 | 1 | 0 | 0.5890 | 0.5576 | 0.6222 |
| factor(X23.CS2)=0 | 2.53 | 489 | 0 | 1 | 0.5890 | 0.5576 | 0.6222 |
| factor(X23.CS2)=0 | 2.55 | 488 | 0 | 1 | 0.5890 | 0.5576 | 0.6222 |
| factor(X23.CS2)=0 | 2.56 | 487 | 0 | 1 | 0.5890 | 0.5576 | 0.6222 |
| factor(X23.CS2)=0 | 2.6 | 486 | 0 | 1 | 0.5890 | 0.5576 | 0.6222 |
| factor(X23.CS2)=0 | 2.61 | 485 | 1 | 1 | 0.5878 | 0.5564 | 0.6210 |
| factor(X23.CS2)=0 | 2.62 | 483 | 2 | 0 | 0.5854 | 0.5539 | 0.6186 |
| factor(X23.CS2)=0 | 2.63 | 481 | 0 | 1 | 0.5854 | 0.5539 | 0.6186 |
| factor(X23.CS2)=0 | 2.64 | 480 | 0 | 1 | 0.5854 | 0.5539 | 0.6186 |
| factor(X23.CS2)=0 | 2.68 | 479 | 0 | 1 | 0.5854 | 0.5539 | 0.6186 |
| factor(X23.CS2)=0 | 2.7 | 478 | 1 | 1 | 0.5841 | 0.5526 | 0.6174 |
| factor(X23.CS2)=0 | 2.73 | 476 | 0 | 1 | 0.5841 | 0.5526 | 0.6174 |
| factor(X23.CS2)=0 | 2.75 | 475 | 0 | 1 | 0.5841 | 0.5526 | 0.6174 |
| factor(X23.CS2)=0 | 2.78 | 474 | 0 | 2 | 0.5841 | 0.5526 | 0.6174 |
| factor(X23.CS2)=0 | 2.79 | 472 | 0 | 2 | 0.5841 | 0.5526 | 0.6174 |
| factor(X23.CS2)=0 | 2.83 | 470 | 0 | 1 | 0.5841 | 0.5526 | 0.6174 |
| factor(X23.CS2)=0 | 2.89 | 469 | 0 | 1 | 0.5841 | 0.5526 | 0.6174 |
| factor(X23.CS2)=0 | 2.93 | 468 | 0 | 1 | 0.5841 | 0.5526 | 0.6174 |
| factor(X23.CS2)=0 | 2.98 | 467 | 0 | 1 | 0.5841 | 0.5526 | 0.6174 |
| factor(X23.CS2)=0 | 3.01 | 466 | 0 | 1 | 0.5841 | 0.5526 | 0.6174 |
| factor(X23.CS2)=0 | 3.02 | 465 | 0 | 1 | 0.5841 | 0.5526 | 0.6174 |
| factor(X23.CS2)=0 | 3.03 | 464 | 0 | 1 | 0.5841 | 0.5526 | 0.6174 |
| factor(X23.CS2)=0 | 3.04 | 463 | 0 | 2 | 0.5841 | 0.5526 | 0.6174 |
| factor(X23.CS2)=0 | 3.05 | 461 | 0 | 1 | 0.5841 | 0.5526 | 0.6174 |
| factor(X23.CS2)=0 | 3.07 | 460 | 0 | 1 | 0.5841 | 0.5526 | 0.6174 |
| factor(X23.CS2)=0 | 3.11 | 459 | 0 | 1 | 0.5841 | 0.5526 | 0.6174 |
| factor(X23.CS2)=0 | 3.14 | 458 | 0 | 1 | 0.5841 | 0.5526 | 0.6174 |
| factor(X23.CS2)=0 | 3.15 | 457 | 0 | 1 | 0.5841 | 0.5526 | 0.6174 |
| factor(X23.CS2)=0 | 3.16 | 456 | 0 | 2 | 0.5841 | 0.5526 | 0.6174 |
| factor(X23.CS2)=0 | 3.17 | 454 | 0 | 1 | 0.5841 | 0.5526 | 0.6174 |
| factor(X23.CS2)=0 | 3.18 | 453 | 1 | 2 | 0.5829 | 0.5513 | 0.6162 |
| factor(X23.CS2)=0 | 3.19 | 450 | 0 | 1 | 0.5829 | 0.5513 | 0.6162 |
| factor(X23.CS2)=0 | 3.2 | 449 | 0 | 1 | 0.5829 | 0.5513 | 0.6162 |
| factor(X23.CS2)=0 | 3.22 | 448 | 0 | 1 | 0.5829 | 0.5513 | 0.6162 |
| factor(X23.CS2)=0 | 3.23 | 447 | 0 | 1 | 0.5829 | 0.5513 | 0.6162 |
| factor(X23.CS2)=0 | 3.24 | 446 | 0 | 1 | 0.5829 | 0.5513 | 0.6162 |
| factor(X23.CS2)=0 | 3.25 | 445 | 0 | 1 | 0.5829 | 0.5513 | 0.6162 |
| factor(X23.CS2)=0 | 3.28 | 444 | 0 | 3 | 0.5829 | 0.5513 | 0.6162 |
| factor(X23.CS2)=0 | 3.29 | 441 | 0 | 1 | 0.5829 | 0.5513 | 0.6162 |
| factor(X23.CS2)=0 | 3.3 | 440 | 0 | 2 | 0.5829 | 0.5513 | 0.6162 |
| factor(X23.CS2)=0 | 3.32 | 438 | 0 | 1 | 0.5829 | 0.5513 | 0.6162 |
| factor(X23.CS2)=0 | 3.33 | 437 | 0 | 3 | 0.5829 | 0.5513 | 0.6162 |
| factor(X23.CS2)=0 | 3.34 | 434 | 0 | 2 | 0.5829 | 0.5513 | 0.6162 |
| factor(X23.CS2)=0 | 3.35 | 432 | 0 | 3 | 0.5829 | 0.5513 | 0.6162 |
| factor(X23.CS2)=0 | 3.36 | 429 | 0 | 2 | 0.5829 | 0.5513 | 0.6162 |
| factor(X23.CS2)=0 | 3.38 | 427 | 0 | 3 | 0.5829 | 0.5513 | 0.6162 |
| factor(X23.CS2)=0 | 3.39 | 424 | 1 | 1 | 0.5815 | 0.5499 | 0.6148 |
| factor(X23.CS2)=0 | 3.4 | 422 | 0 | 1 | 0.5815 | 0.5499 | 0.6148 |
| factor(X23.CS2)=0 | 3.41 | 421 | 0 | 4 | 0.5815 | 0.5499 | 0.6148 |
| factor(X23.CS2)=0 | 3.43 | 417 | 0 | 1 | 0.5815 | 0.5499 | 0.6148 |
| factor(X23.CS2)=0 | 3.44 | 416 | 0 | 4 | 0.5815 | 0.5499 | 0.6148 |
| factor(X23.CS2)=0 | 3.45 | 412 | 0 | 2 | 0.5815 | 0.5499 | 0.6148 |
| factor(X23.CS2)=0 | 3.46 | 410 | 0 | 1 | 0.5815 | 0.5499 | 0.6148 |
| factor(X23.CS2)=0 | 3.48 | 409 | 0 | 2 | 0.5815 | 0.5499 | 0.6148 |
| factor(X23.CS2)=0 | 3.5 | 407 | 0 | 3 | 0.5815 | 0.5499 | 0.6148 |
| factor(X23.CS2)=0 | 3.52 | 404 | 0 | 1 | 0.5815 | 0.5499 | 0.6148 |
| factor(X23.CS2)=0 | 3.54 | 403 | 1 | 1 | 0.5800 | 0.5484 | 0.6134 |
| factor(X23.CS2)=0 | 3.56 | 401 | 0 | 4 | 0.5800 | 0.5484 | 0.6134 |
| factor(X23.CS2)=0 | 3.58 | 397 | 0 | 1 | 0.5800 | 0.5484 | 0.6134 |
| factor(X23.CS2)=0 | 3.62 | 396 | 0 | 1 | 0.5800 | 0.5484 | 0.6134 |
| factor(X23.CS2)=0 | 3.63 | 395 | 0 | 1 | 0.5800 | 0.5484 | 0.6134 |
| factor(X23.CS2)=0 | 3.65 | 394 | 0 | 2 | 0.5800 | 0.5484 | 0.6134 |
| factor(X23.CS2)=0 | 3.66 | 392 | 0 | 1 | 0.5800 | 0.5484 | 0.6134 |
| factor(X23.CS2)=0 | 3.74 | 391 | 0 | 1 | 0.5800 | 0.5484 | 0.6134 |
| factor(X23.CS2)=0 | 3.79 | 390 | 0 | 1 | 0.5800 | 0.5484 | 0.6134 |
| factor(X23.CS2)=0 | 3.81 | 389 | 0 | 2 | 0.5800 | 0.5484 | 0.6134 |
| factor(X23.CS2)=0 | 3.83 | 387 | 0 | 1 | 0.5800 | 0.5484 | 0.6134 |
| factor(X23.CS2)=0 | 3.84 | 386 | 0 | 2 | 0.5800 | 0.5484 | 0.6134 |
| factor(X23.CS2)=0 | 3.89 | 384 | 0 | 1 | 0.5800 | 0.5484 | 0.6134 |
| factor(X23.CS2)=0 | 3.9 | 383 | 0 | 2 | 0.5800 | 0.5484 | 0.6134 |
| factor(X23.CS2)=0 | 3.95 | 381 | 0 | 1 | 0.5800 | 0.5484 | 0.6134 |
| factor(X23.CS2)=0 | 3.96 | 380 | 0 | 1 | 0.5800 | 0.5484 | 0.6134 |
| factor(X23.CS2)=0 | 3.97 | 379 | 0 | 1 | 0.5800 | 0.5484 | 0.6134 |
| factor(X23.CS2)=0 | 3.99 | 378 | 1 | 0 | 0.5785 | 0.5469 | 0.6120 |
| factor(X23.CS2)=0 | 4.04 | 377 | 0 | 1 | 0.5785 | 0.5469 | 0.6120 |
| factor(X23.CS2)=0 | 4.05 | 376 | 0 | 2 | 0.5785 | 0.5469 | 0.6120 |
| factor(X23.CS2)=0 | 4.06 | 374 | 0 | 2 | 0.5785 | 0.5469 | 0.6120 |
| factor(X23.CS2)=0 | 4.07 | 372 | 1 | 1 | 0.5769 | 0.5453 | 0.6105 |
| factor(X23.CS2)=0 | 4.08 | 370 | 0 | 2 | 0.5769 | 0.5453 | 0.6105 |
| factor(X23.CS2)=0 | 4.13 | 368 | 0 | 2 | 0.5769 | 0.5453 | 0.6105 |
| factor(X23.CS2)=0 | 4.15 | 366 | 0 | 2 | 0.5769 | 0.5453 | 0.6105 |
| factor(X23.CS2)=0 | 4.19 | 364 | 0 | 2 | 0.5769 | 0.5453 | 0.6105 |
| factor(X23.CS2)=0 | 4.22 | 362 | 0 | 1 | 0.5769 | 0.5453 | 0.6105 |
| factor(X23.CS2)=0 | 4.23 | 361 | 1 | 0 | 0.5754 | 0.5436 | 0.6089 |
| factor(X23.CS2)=0 | 4.26 | 360 | 0 | 1 | 0.5754 | 0.5436 | 0.6089 |
| factor(X23.CS2)=0 | 4.27 | 359 | 0 | 2 | 0.5754 | 0.5436 | 0.6089 |
| factor(X23.CS2)=0 | 4.31 | 357 | 0 | 2 | 0.5754 | 0.5436 | 0.6089 |
| factor(X23.CS2)=0 | 4.32 | 355 | 0 | 1 | 0.5754 | 0.5436 | 0.6089 |
| factor(X23.CS2)=0 | 4.34 | 354 | 0 | 1 | 0.5754 | 0.5436 | 0.6089 |
| factor(X23.CS2)=0 | 4.35 | 353 | 0 | 4 | 0.5754 | 0.5436 | 0.6089 |
| factor(X23.CS2)=0 | 4.36 | 349 | 0 | 4 | 0.5754 | 0.5436 | 0.6089 |
| factor(X23.CS2)=0 | 4.38 | 345 | 0 | 3 | 0.5754 | 0.5436 | 0.6089 |
| factor(X23.CS2)=0 | 4.4 | 342 | 0 | 1 | 0.5754 | 0.5436 | 0.6089 |
| factor(X23.CS2)=0 | 4.41 | 341 | 0 | 1 | 0.5754 | 0.5436 | 0.6089 |
| factor(X23.CS2)=0 | 4.42 | 340 | 0 | 2 | 0.5754 | 0.5436 | 0.6089 |
| factor(X23.CS2)=0 | 4.44 | 338 | 0 | 2 | 0.5754 | 0.5436 | 0.6089 |
| factor(X23.CS2)=0 | 4.45 | 336 | 0 | 1 | 0.5754 | 0.5436 | 0.6089 |
| factor(X23.CS2)=0 | 4.47 | 335 | 0 | 1 | 0.5754 | 0.5436 | 0.6089 |
| factor(X23.CS2)=0 | 4.48 | 334 | 0 | 1 | 0.5754 | 0.5436 | 0.6089 |
| factor(X23.CS2)=0 | 4.49 | 333 | 0 | 2 | 0.5754 | 0.5436 | 0.6089 |
| factor(X23.CS2)=0 | 4.5 | 331 | 0 | 1 | 0.5754 | 0.5436 | 0.6089 |
| factor(X23.CS2)=0 | 4.53 | 330 | 0 | 1 | 0.5754 | 0.5436 | 0.6089 |
| factor(X23.CS2)=0 | 4.54 | 329 | 0 | 1 | 0.5754 | 0.5436 | 0.6089 |
| factor(X23.CS2)=0 | 4.58 | 328 | 0 | 2 | 0.5754 | 0.5436 | 0.6089 |
| factor(X23.CS2)=0 | 4.59 | 326 | 0 | 1 | 0.5754 | 0.5436 | 0.6089 |
| factor(X23.CS2)=0 | 4.6 | 325 | 0 | 3 | 0.5754 | 0.5436 | 0.6089 |
| factor(X23.CS2)=0 | 4.61 | 322 | 0 | 3 | 0.5754 | 0.5436 | 0.6089 |
| factor(X23.CS2)=0 | 4.62 | 319 | 0 | 1 | 0.5754 | 0.5436 | 0.6089 |
| factor(X23.CS2)=0 | 4.64 | 318 | 0 | 1 | 0.5754 | 0.5436 | 0.6089 |
| factor(X23.CS2)=0 | 4.72 | 317 | 0 | 1 | 0.5754 | 0.5436 | 0.6089 |
| factor(X23.CS2)=0 | 4.73 | 316 | 0 | 2 | 0.5754 | 0.5436 | 0.6089 |
| factor(X23.CS2)=0 | 4.75 | 314 | 0 | 1 | 0.5754 | 0.5436 | 0.6089 |
| factor(X23.CS2)=0 | 4.79 | 313 | 0 | 1 | 0.5754 | 0.5436 | 0.6089 |
| factor(X23.CS2)=0 | 4.81 | 312 | 0 | 1 | 0.5754 | 0.5436 | 0.6089 |
| factor(X23.CS2)=0 | 4.82 | 311 | 0 | 1 | 0.5754 | 0.5436 | 0.6089 |
| factor(X23.CS2)=0 | 4.85 | 310 | 0 | 4 | 0.5754 | 0.5436 | 0.6089 |
| factor(X23.CS2)=0 | 4.87 | 306 | 0 | 2 | 0.5754 | 0.5436 | 0.6089 |
| factor(X23.CS2)=0 | 4.9 | 304 | 0 | 1 | 0.5754 | 0.5436 | 0.6089 |
| factor(X23.CS2)=0 | 4.91 | 303 | 0 | 1 | 0.5754 | 0.5436 | 0.6089 |
| factor(X23.CS2)=0 | 4.94 | 302 | 0 | 2 | 0.5754 | 0.5436 | 0.6089 |
| factor(X23.CS2)=0 | 4.97 | 300 | 0 | 1 | 0.5754 | 0.5436 | 0.6089 |
| factor(X23.CS2)=0 | 4.99 | 299 | 0 | 1 | 0.5754 | 0.5436 | 0.6089 |
| factor(X23.CS2)=0 | 5 | 298 | 0 | 298 | 0.5754 | 0.5436 | 0.6089 |
| factor(X23.CS2)=1 | 0.41 | 18 | 2 | 0 | 0.8889 | 0.7549 | 1.0000 |
| factor(X23.CS2)=1 | 0.7 | 16 | 2 | 0 | 0.7778 | 0.6076 | 0.9956 |
| factor(X23.CS2)=1 | 0.76 | 14 | 2 | 0 | 0.6667 | 0.4809 | 0.9242 |
| factor(X23.CS2)=1 | 1.06 | 12 | 1 | 0 | 0.6111 | 0.4227 | 0.8834 |
| factor(X23.CS2)=1 | 1.08 | 11 | 1 | 0 | 0.5556 | 0.3675 | 0.8398 |
| factor(X23.CS2)=1 | 1.09 | 10 | 1 | 0 | 0.5000 | 0.3150 | 0.7936 |
| factor(X23.CS2)=1 | 1.31 | 9 | 2 | 0 | 0.3889 | 0.2179 | 0.6940 |
| factor(X23.CS2)=1 | 1.46 | 7 | 1 | 0 | 0.3333 | 0.1734 | 0.6406 |
| factor(X23.CS2)=1 | 3.39 | 6 | 0 | 1 | 0.3333 | 0.1734 | 0.6406 |
| factor(X23.CS2)=1 | 4.33 | 5 | 0 | 1 | 0.3333 | 0.1734 | 0.6406 |
| factor(X23.CS2)=1 | 4.55 | 4 | 1 | 0 | 0.2500 | 0.1053 | 0.5933 |
| factor(X23.CS2)=1 | 4.68 | 3 | 1 | 0 | 0.1667 | 0.0513 | 0.5412 |
| factor(X23.CS2)=1 | 4.72 | 2 | 1 | 0 | 0.0833 | 0.0135 | 0.5137 |
| factor(X23.CS2)=1 | 5 | 1 | 0 | 1 | 0.0833 | 0.0135 | 0.5137 |
| factor(X23.CS2)=2 | 0.13 | 281 | 2 | 0 | 0.9929 | 0.9831 | 1.0000 |
| factor(X23.CS2)=2 | 0.15 | 279 | 2 | 0 | 0.9858 | 0.9720 | 0.9997 |
| factor(X23.CS2)=2 | 0.16 | 277 | 5 | 0 | 0.9680 | 0.9476 | 0.9888 |
| factor(X23.CS2)=2 | 0.17 | 272 | 3 | 0 | 0.9573 | 0.9339 | 0.9812 |
| factor(X23.CS2)=2 | 0.18 | 269 | 4 | 0 | 0.9431 | 0.9164 | 0.9705 |
| factor(X23.CS2)=2 | 0.19 | 265 | 11 | 0 | 0.9039 | 0.8701 | 0.9390 |
| factor(X23.CS2)=2 | 0.2 | 254 | 4 | 0 | 0.8897 | 0.8538 | 0.9271 |
| factor(X23.CS2)=2 | 0.21 | 250 | 11 | 0 | 0.8505 | 0.8099 | 0.8933 |
| factor(X23.CS2)=2 | 0.22 | 239 | 10 | 0 | 0.8149 | 0.7708 | 0.8616 |
| factor(X23.CS2)=2 | 0.23 | 229 | 8 | 0 | 0.7865 | 0.7400 | 0.8359 |
| factor(X23.CS2)=2 | 0.24 | 221 | 3 | 0 | 0.7758 | 0.7285 | 0.8261 |
| factor(X23.CS2)=2 | 0.25 | 218 | 3 | 0 | 0.7651 | 0.7171 | 0.8163 |
| factor(X23.CS2)=2 | 0.27 | 215 | 2 | 0 | 0.7580 | 0.7095 | 0.8098 |
| factor(X23.CS2)=2 | 0.28 | 213 | 3 | 0 | 0.7473 | 0.6982 | 0.7999 |
| factor(X23.CS2)=2 | 0.29 | 210 | 1 | 0 | 0.7438 | 0.6944 | 0.7966 |
| factor(X23.CS2)=2 | 0.3 | 209 | 3 | 0 | 0.7331 | 0.6832 | 0.7867 |
| factor(X23.CS2)=2 | 0.32 | 206 | 2 | 0 | 0.7260 | 0.6757 | 0.7800 |
| factor(X23.CS2)=2 | 0.33 | 204 | 1 | 1 | 0.7224 | 0.6719 | 0.7767 |
| factor(X23.CS2)=2 | 0.34 | 202 | 1 | 0 | 0.7188 | 0.6682 | 0.7734 |
| factor(X23.CS2)=2 | 0.35 | 201 | 2 | 0 | 0.7117 | 0.6606 | 0.7667 |
| factor(X23.CS2)=2 | 0.36 | 199 | 1 | 0 | 0.7081 | 0.6569 | 0.7633 |
| factor(X23.CS2)=2 | 0.37 | 198 | 1 | 0 | 0.7045 | 0.6532 | 0.7600 |
| factor(X23.CS2)=2 | 0.42 | 197 | 1 | 0 | 0.7010 | 0.6494 | 0.7566 |
| factor(X23.CS2)=2 | 0.43 | 196 | 1 | 0 | 0.6974 | 0.6457 | 0.7532 |
| factor(X23.CS2)=2 | 0.45 | 195 | 1 | 0 | 0.6938 | 0.6419 | 0.7499 |
| factor(X23.CS2)=2 | 0.47 | 194 | 2 | 0 | 0.6867 | 0.6345 | 0.7431 |
| factor(X23.CS2)=2 | 0.48 | 192 | 2 | 0 | 0.6795 | 0.6271 | 0.7363 |
| factor(X23.CS2)=2 | 0.49 | 190 | 3 | 0 | 0.6688 | 0.6159 | 0.7262 |
| factor(X23.CS2)=2 | 0.5 | 187 | 1 | 0 | 0.6652 | 0.6122 | 0.7228 |
| factor(X23.CS2)=2 | 0.52 | 186 | 2 | 0 | 0.6580 | 0.6048 | 0.7160 |
| factor(X23.CS2)=2 | 0.53 | 184 | 3 | 0 | 0.6473 | 0.5938 | 0.7057 |
| factor(X23.CS2)=2 | 0.54 | 181 | 1 | 0 | 0.6437 | 0.5901 | 0.7023 |
| factor(X23.CS2)=2 | 0.55 | 180 | 2 | 0 | 0.6366 | 0.5827 | 0.6954 |
| factor(X23.CS2)=2 | 0.56 | 178 | 1 | 0 | 0.6330 | 0.5790 | 0.6920 |
| factor(X23.CS2)=2 | 0.57 | 177 | 1 | 0 | 0.6294 | 0.5754 | 0.6886 |
| factor(X23.CS2)=2 | 0.58 | 176 | 1 | 0 | 0.6259 | 0.5717 | 0.6851 |
| factor(X23.CS2)=2 | 0.59 | 175 | 1 | 0 | 0.6223 | 0.5681 | 0.6817 |
| factor(X23.CS2)=2 | 0.6 | 174 | 1 | 0 | 0.6187 | 0.5644 | 0.6782 |
| factor(X23.CS2)=2 | 0.62 | 173 | 1 | 0 | 0.6151 | 0.5607 | 0.6748 |
| factor(X23.CS2)=2 | 0.64 | 172 | 1 | 0 | 0.6116 | 0.5571 | 0.6713 |
| factor(X23.CS2)=2 | 0.66 | 171 | 1 | 0 | 0.6080 | 0.5534 | 0.6679 |
| factor(X23.CS2)=2 | 0.68 | 170 | 3 | 0 | 0.5972 | 0.5425 | 0.6575 |
| factor(X23.CS2)=2 | 0.69 | 167 | 2 | 0 | 0.5901 | 0.5352 | 0.6506 |
| factor(X23.CS2)=2 | 0.7 | 165 | 2 | 0 | 0.5829 | 0.5280 | 0.6436 |
| factor(X23.CS2)=2 | 0.71 | 163 | 1 | 0 | 0.5794 | 0.5244 | 0.6401 |
| factor(X23.CS2)=2 | 0.72 | 162 | 3 | 0 | 0.5686 | 0.5135 | 0.6297 |
| factor(X23.CS2)=2 | 0.74 | 159 | 2 | 0 | 0.5615 | 0.5063 | 0.6227 |
| factor(X23.CS2)=2 | 0.75 | 157 | 2 | 0 | 0.5543 | 0.4991 | 0.6157 |
| factor(X23.CS2)=2 | 0.77 | 155 | 2 | 0 | 0.5472 | 0.4919 | 0.6087 |
| factor(X23.CS2)=2 | 0.78 | 153 | 3 | 1 | 0.5365 | 0.4811 | 0.5981 |
| factor(X23.CS2)=2 | 0.79 | 149 | 2 | 0 | 0.5292 | 0.4739 | 0.5910 |
| factor(X23.CS2)=2 | 0.81 | 147 | 3 | 0 | 0.5184 | 0.4631 | 0.5804 |
| factor(X23.CS2)=2 | 0.82 | 144 | 3 | 0 | 0.5076 | 0.4523 | 0.5697 |
| factor(X23.CS2)=2 | 0.84 | 141 | 4 | 0 | 0.4932 | 0.4380 | 0.5554 |
| factor(X23.CS2)=2 | 0.85 | 137 | 1 | 0 | 0.4896 | 0.4344 | 0.5519 |
| factor(X23.CS2)=2 | 0.86 | 136 | 1 | 1 | 0.4860 | 0.4309 | 0.5483 |
| factor(X23.CS2)=2 | 0.87 | 134 | 2 | 0 | 0.4788 | 0.4237 | 0.5411 |
| factor(X23.CS2)=2 | 0.88 | 132 | 1 | 0 | 0.4752 | 0.4201 | 0.5375 |
| factor(X23.CS2)=2 | 0.9 | 131 | 1 | 0 | 0.4715 | 0.4165 | 0.5338 |
| factor(X23.CS2)=2 | 0.91 | 130 | 4 | 0 | 0.4570 | 0.4022 | 0.5193 |
| factor(X23.CS2)=2 | 0.92 | 126 | 1 | 0 | 0.4534 | 0.3986 | 0.5157 |
| factor(X23.CS2)=2 | 0.93 | 125 | 1 | 0 | 0.4498 | 0.3950 | 0.5121 |
| factor(X23.CS2)=2 | 0.95 | 124 | 1 | 0 | 0.4461 | 0.3915 | 0.5084 |
| factor(X23.CS2)=2 | 0.97 | 123 | 2 | 0 | 0.4389 | 0.3844 | 0.5012 |
| factor(X23.CS2)=2 | 0.98 | 121 | 1 | 0 | 0.4353 | 0.3808 | 0.4975 |
| factor(X23.CS2)=2 | 0.99 | 120 | 2 | 0 | 0.4280 | 0.3737 | 0.4902 |
| factor(X23.CS2)=2 | 1 | 118 | 1 | 0 | 0.4244 | 0.3702 | 0.4865 |
| factor(X23.CS2)=2 | 1.02 | 117 | 1 | 0 | 0.4208 | 0.3666 | 0.4829 |
| factor(X23.CS2)=2 | 1.07 | 116 | 1 | 0 | 0.4171 | 0.3631 | 0.4792 |
| factor(X23.CS2)=2 | 1.08 | 115 | 2 | 0 | 0.4099 | 0.3560 | 0.4719 |
| factor(X23.CS2)=2 | 1.11 | 113 | 1 | 0 | 0.4062 | 0.3525 | 0.4682 |
| factor(X23.CS2)=2 | 1.12 | 112 | 1 | 0 | 0.4026 | 0.3490 | 0.4645 |
| factor(X23.CS2)=2 | 1.15 | 111 | 2 | 0 | 0.3954 | 0.3419 | 0.4572 |
| factor(X23.CS2)=2 | 1.16 | 109 | 1 | 0 | 0.3917 | 0.3384 | 0.4535 |
| factor(X23.CS2)=2 | 1.17 | 108 | 2 | 0 | 0.3845 | 0.3314 | 0.4461 |
| factor(X23.CS2)=2 | 1.18 | 106 | 1 | 0 | 0.3809 | 0.3279 | 0.4424 |
| factor(X23.CS2)=2 | 1.2 | 105 | 2 | 0 | 0.3736 | 0.3209 | 0.4350 |
| factor(X23.CS2)=2 | 1.25 | 103 | 2 | 0 | 0.3663 | 0.3139 | 0.4276 |
| factor(X23.CS2)=2 | 1.3 | 101 | 1 | 0 | 0.3627 | 0.3104 | 0.4239 |
| factor(X23.CS2)=2 | 1.35 | 100 | 2 | 0 | 0.3555 | 0.3034 | 0.4164 |
| factor(X23.CS2)=2 | 1.36 | 98 | 1 | 0 | 0.3518 | 0.3000 | 0.4127 |
| factor(X23.CS2)=2 | 1.38 | 97 | 2 | 0 | 0.3446 | 0.2930 | 0.4052 |
| factor(X23.CS2)=2 | 1.39 | 95 | 2 | 1 | 0.3373 | 0.2861 | 0.3978 |
| factor(X23.CS2)=2 | 1.42 | 92 | 1 | 0 | 0.3337 | 0.2826 | 0.3940 |
| factor(X23.CS2)=2 | 1.45 | 91 | 1 | 0 | 0.3300 | 0.2791 | 0.3902 |
| factor(X23.CS2)=2 | 1.5 | 90 | 2 | 0 | 0.3227 | 0.2721 | 0.3826 |
| factor(X23.CS2)=2 | 1.55 | 88 | 2 | 0 | 0.3153 | 0.2651 | 0.3750 |
| factor(X23.CS2)=2 | 1.58 | 86 | 1 | 0 | 0.3117 | 0.2616 | 0.3712 |
| factor(X23.CS2)=2 | 1.72 | 85 | 1 | 0 | 0.3080 | 0.2582 | 0.3674 |
| factor(X23.CS2)=2 | 1.74 | 84 | 1 | 0 | 0.3043 | 0.2547 | 0.3636 |
| factor(X23.CS2)=2 | 1.79 | 83 | 1 | 0 | 0.3007 | 0.2512 | 0.3598 |
| factor(X23.CS2)=2 | 1.92 | 82 | 1 | 0 | 0.2970 | 0.2478 | 0.3560 |
| factor(X23.CS2)=2 | 1.98 | 81 | 4 | 0 | 0.2823 | 0.2340 | 0.3407 |
| factor(X23.CS2)=2 | 2.09 | 77 | 1 | 0 | 0.2787 | 0.2305 | 0.3368 |
| factor(X23.CS2)=2 | 2.26 | 76 | 1 | 0 | 0.2750 | 0.2271 | 0.3330 |
| factor(X23.CS2)=2 | 2.32 | 75 | 0 | 1 | 0.2750 | 0.2271 | 0.3330 |
| factor(X23.CS2)=2 | 2.53 | 74 | 1 | 0 | 0.2713 | 0.2236 | 0.3291 |
| factor(X23.CS2)=2 | 2.71 | 73 | 0 | 1 | 0.2713 | 0.2236 | 0.3291 |
| factor(X23.CS2)=2 | 2.72 | 72 | 3 | 0 | 0.2600 | 0.2130 | 0.3173 |
| factor(X23.CS2)=2 | 2.83 | 69 | 1 | 0 | 0.2562 | 0.2095 | 0.3133 |
| factor(X23.CS2)=2 | 2.95 | 68 | 0 | 1 | 0.2562 | 0.2095 | 0.3133 |
| factor(X23.CS2)=2 | 2.99 | 67 | 1 | 0 | 0.2524 | 0.2060 | 0.3093 |
| factor(X23.CS2)=2 | 3 | 66 | 0 | 1 | 0.2524 | 0.2060 | 0.3093 |
| factor(X23.CS2)=2 | 3.05 | 65 | 0 | 1 | 0.2524 | 0.2060 | 0.3093 |
| factor(X23.CS2)=2 | 3.18 | 64 | 0 | 1 | 0.2524 | 0.2060 | 0.3093 |
| factor(X23.CS2)=2 | 3.25 | 63 | 0 | 1 | 0.2524 | 0.2060 | 0.3093 |
| factor(X23.CS2)=2 | 3.39 | 62 | 2 | 0 | 0.2442 | 0.1983 | 0.3008 |
| factor(X23.CS2)=2 | 3.53 | 60 | 0 | 1 | 0.2442 | 0.1983 | 0.3008 |
| factor(X23.CS2)=2 | 3.68 | 59 | 0 | 1 | 0.2442 | 0.1983 | 0.3008 |
| factor(X23.CS2)=2 | 3.87 | 58 | 0 | 1 | 0.2442 | 0.1983 | 0.3008 |
| factor(X23.CS2)=2 | 3.88 | 57 | 0 | 1 | 0.2442 | 0.1983 | 0.3008 |
| factor(X23.CS2)=2 | 4.04 | 56 | 0 | 1 | 0.2442 | 0.1983 | 0.3008 |
| factor(X23.CS2)=2 | 4.16 | 55 | 0 | 1 | 0.2442 | 0.1983 | 0.3008 |
| factor(X23.CS2)=2 | 4.22 | 54 | 0 | 1 | 0.2442 | 0.1983 | 0.3008 |
| factor(X23.CS2)=2 | 4.41 | 53 | 0 | 1 | 0.2442 | 0.1983 | 0.3008 |
| factor(X23.CS2)=2 | 4.44 | 52 | 0 | 1 | 0.2442 | 0.1983 | 0.3008 |
| factor(X23.CS2)=2 | 4.47 | 51 | 0 | 1 | 0.2442 | 0.1983 | 0.3008 |
| factor(X23.CS2)=2 | 4.48 | 50 | 0 | 1 | 0.2442 | 0.1983 | 0.3008 |
| factor(X23.CS2)=2 | 4.53 | 49 | 0 | 2 | 0.2442 | 0.1983 | 0.3008 |
| factor(X23.CS2)=2 | 4.55 | 47 | 0 | 1 | 0.2442 | 0.1983 | 0.3008 |
| factor(X23.CS2)=2 | 4.6 | 46 | 0 | 1 | 0.2442 | 0.1983 | 0.3008 |
| factor(X23.CS2)=2 | 4.61 | 45 | 0 | 1 | 0.2442 | 0.1983 | 0.3008 |
| factor(X23.CS2)=2 | 4.65 | 44 | 0 | 1 | 0.2442 | 0.1983 | 0.3008 |
| factor(X23.CS2)=2 | 4.7 | 43 | 0 | 1 | 0.2442 | 0.1983 | 0.3008 |
| factor(X23.CS2)=2 | 4.81 | 42 | 0 | 1 | 0.2442 | 0.1983 | 0.3008 |
| factor(X23.CS2)=2 | 4.88 | 41 | 0 | 1 | 0.2442 | 0.1983 | 0.3008 |
| factor(X23.CS2)=2 | 4.89 | 40 | 0 | 1 | 0.2442 | 0.1983 | 0.3008 |
| factor(X23.CS2)=2 | 5 | 39 | 0 | 39 | 0.2442 | 0.1983 | 0.3008 |

|  |  |  |  |  |  |  |  |  |  |
| --- | --- | --- | --- | --- | --- | --- | --- | --- | --- |
| X23.CS2 | records | n.max | n.start | events | \*rmean | \*se(rmean) | median | 0.95LCL | 0.95UCL |
| factor(X23.CS2)=0 | 906 | 906 | 906 | 378 | 3.318 | 0.067 | NA | NA | NA |
| factor(X23.CS2)=1 | 18 | 18 | 18 | 15 | 2.193 | 0.43 | 1.2 | 0.76 | NA |
| factor(X23.CS2)=2 | 281 | 281 | 281 | 210 | 1.796 | 0.115 | 0.84 | 0.75 | 0.98 |

Landmark analysis for time segment: EFS(years) > 5
Log rank test: implements the G-rho family of Harrington and Fleming (1982), with weights on each death of S(t)^rho, where S is the Kaplan-Meier estimate of survival. With rho = 0 this is the log-rank or Mantel-Haenszel test.

|  |  |  |  |
| --- | --- | --- | --- |
|  | N | Observed | Expected |
| factor(X23.CS2)=0 | 297 | 5 | 6.9635 |
| factor(X23.CS2)=1 | 1 | 1 | 0.0376 |
| factor(X23.CS2)=2 | 39 | 2 | 0.9989 |

Chisq=
26.4054
on
2
degree of freedom, p=
<0.0001
Survival table

|  |  |  |  |  |  |  |  |
| --- | --- | --- | --- | --- | --- | --- | --- |
|  | EFS(years) | N.Risk | N.Event | N.Censor | Survival | 95%CI Low | 95%CI Upp |
| factor(X23.CS2)=0 | 5.02 | 297 | 0 | 1 | 1.0000 | 1.0000 | 1.0000 |
| factor(X23.CS2)=0 | 5.03 | 296 | 0 | 1 | 1.0000 | 1.0000 | 1.0000 |
| factor(X23.CS2)=0 | 5.04 | 295 | 0 | 1 | 1.0000 | 1.0000 | 1.0000 |
| factor(X23.CS2)=0 | 5.05 | 294 | 0 | 2 | 1.0000 | 1.0000 | 1.0000 |
| factor(X23.CS2)=0 | 5.08 | 292 | 0 | 1 | 1.0000 | 1.0000 | 1.0000 |
| factor(X23.CS2)=0 | 5.1 | 291 | 0 | 1 | 1.0000 | 1.0000 | 1.0000 |
| factor(X23.CS2)=0 | 5.12 | 290 | 0 | 1 | 1.0000 | 1.0000 | 1.0000 |
| factor(X23.CS2)=0 | 5.14 | 289 | 0 | 1 | 1.0000 | 1.0000 | 1.0000 |
| factor(X23.CS2)=0 | 5.15 | 288 | 0 | 2 | 1.0000 | 1.0000 | 1.0000 |
| factor(X23.CS2)=0 | 5.16 | 286 | 0 | 1 | 1.0000 | 1.0000 | 1.0000 |
| factor(X23.CS2)=0 | 5.17 | 285 | 0 | 1 | 1.0000 | 1.0000 | 1.0000 |
| factor(X23.CS2)=0 | 5.18 | 284 | 0 | 3 | 1.0000 | 1.0000 | 1.0000 |
| factor(X23.CS2)=0 | 5.2 | 281 | 0 | 1 | 1.0000 | 1.0000 | 1.0000 |
| factor(X23.CS2)=0 | 5.22 | 280 | 0 | 1 | 1.0000 | 1.0000 | 1.0000 |
| factor(X23.CS2)=0 | 5.25 | 279 | 0 | 2 | 1.0000 | 1.0000 | 1.0000 |
| factor(X23.CS2)=0 | 5.26 | 277 | 0 | 1 | 1.0000 | 1.0000 | 1.0000 |
| factor(X23.CS2)=0 | 5.27 | 276 | 0 | 2 | 1.0000 | 1.0000 | 1.0000 |
| factor(X23.CS2)=0 | 5.29 | 274 | 0 | 2 | 1.0000 | 1.0000 | 1.0000 |
| factor(X23.CS2)=0 | 5.3 | 272 | 0 | 1 | 1.0000 | 1.0000 | 1.0000 |
| factor(X23.CS2)=0 | 5.31 | 271 | 0 | 1 | 1.0000 | 1.0000 | 1.0000 |
| factor(X23.CS2)=0 | 5.32 | 270 | 0 | 2 | 1.0000 | 1.0000 | 1.0000 |
| factor(X23.CS2)=0 | 5.33 | 268 | 0 | 2 | 1.0000 | 1.0000 | 1.0000 |
| factor(X23.CS2)=0 | 5.34 | 266 | 0 | 1 | 1.0000 | 1.0000 | 1.0000 |
| factor(X23.CS2)=0 | 5.35 | 265 | 0 | 6 | 1.0000 | 1.0000 | 1.0000 |
| factor(X23.CS2)=0 | 5.36 | 259 | 0 | 1 | 1.0000 | 1.0000 | 1.0000 |
| factor(X23.CS2)=0 | 5.37 | 258 | 0 | 2 | 1.0000 | 1.0000 | 1.0000 |
| factor(X23.CS2)=0 | 5.38 | 256 | 0 | 2 | 1.0000 | 1.0000 | 1.0000 |
| factor(X23.CS2)=0 | 5.39 | 254 | 0 | 1 | 1.0000 | 1.0000 | 1.0000 |
| factor(X23.CS2)=0 | 5.4 | 253 | 0 | 1 | 1.0000 | 1.0000 | 1.0000 |
| factor(X23.CS2)=0 | 5.41 | 252 | 0 | 2 | 1.0000 | 1.0000 | 1.0000 |
| factor(X23.CS2)=0 | 5.42 | 250 | 0 | 3 | 1.0000 | 1.0000 | 1.0000 |
| factor(X23.CS2)=0 | 5.43 | 247 | 0 | 1 | 1.0000 | 1.0000 | 1.0000 |
| factor(X23.CS2)=0 | 5.44 | 246 | 0 | 4 | 1.0000 | 1.0000 | 1.0000 |
| factor(X23.CS2)=0 | 5.45 | 242 | 0 | 1 | 1.0000 | 1.0000 | 1.0000 |
| factor(X23.CS2)=0 | 5.46 | 241 | 0 | 2 | 1.0000 | 1.0000 | 1.0000 |
| factor(X23.CS2)=0 | 5.48 | 239 | 0 | 2 | 1.0000 | 1.0000 | 1.0000 |
| factor(X23.CS2)=0 | 5.49 | 237 | 0 | 1 | 1.0000 | 1.0000 | 1.0000 |
| factor(X23.CS2)=0 | 5.5 | 236 | 0 | 1 | 1.0000 | 1.0000 | 1.0000 |
| factor(X23.CS2)=0 | 5.51 | 235 | 0 | 2 | 1.0000 | 1.0000 | 1.0000 |
| factor(X23.CS2)=0 | 5.52 | 233 | 0 | 2 | 1.0000 | 1.0000 | 1.0000 |
| factor(X23.CS2)=0 | 5.54 | 231 | 0 | 4 | 1.0000 | 1.0000 | 1.0000 |
| factor(X23.CS2)=0 | 5.55 | 227 | 0 | 1 | 1.0000 | 1.0000 | 1.0000 |
| factor(X23.CS2)=0 | 5.56 | 226 | 0 | 2 | 1.0000 | 1.0000 | 1.0000 |
| factor(X23.CS2)=0 | 5.57 | 224 | 0 | 1 | 1.0000 | 1.0000 | 1.0000 |
| factor(X23.CS2)=0 | 5.58 | 223 | 1 | 1 | 0.9955 | 0.9868 | 1.0000 |
| factor(X23.CS2)=0 | 5.6 | 221 | 0 | 1 | 0.9955 | 0.9868 | 1.0000 |
| factor(X23.CS2)=0 | 5.61 | 220 | 0 | 1 | 0.9955 | 0.9868 | 1.0000 |
| factor(X23.CS2)=0 | 5.63 | 219 | 0 | 2 | 0.9955 | 0.9868 | 1.0000 |
| factor(X23.CS2)=0 | 5.65 | 217 | 0 | 1 | 0.9955 | 0.9868 | 1.0000 |
| factor(X23.CS2)=0 | 5.66 | 216 | 0 | 1 | 0.9955 | 0.9868 | 1.0000 |
| factor(X23.CS2)=0 | 5.67 | 215 | 0 | 3 | 0.9955 | 0.9868 | 1.0000 |
| factor(X23.CS2)=0 | 5.7 | 212 | 0 | 3 | 0.9955 | 0.9868 | 1.0000 |
| factor(X23.CS2)=0 | 5.71 | 209 | 0 | 2 | 0.9955 | 0.9868 | 1.0000 |
| factor(X23.CS2)=0 | 5.72 | 207 | 0 | 1 | 0.9955 | 0.9868 | 1.0000 |
| factor(X23.CS2)=0 | 5.73 | 206 | 0 | 2 | 0.9955 | 0.9868 | 1.0000 |
| factor(X23.CS2)=0 | 5.74 | 204 | 0 | 1 | 0.9955 | 0.9868 | 1.0000 |
| factor(X23.CS2)=0 | 5.75 | 203 | 0 | 2 | 0.9955 | 0.9868 | 1.0000 |
| factor(X23.CS2)=0 | 5.77 | 201 | 0 | 1 | 0.9955 | 0.9868 | 1.0000 |
| factor(X23.CS2)=0 | 5.78 | 200 | 0 | 2 | 0.9955 | 0.9868 | 1.0000 |
| factor(X23.CS2)=0 | 5.79 | 198 | 0 | 1 | 0.9955 | 0.9868 | 1.0000 |
| factor(X23.CS2)=0 | 5.8 | 197 | 0 | 1 | 0.9955 | 0.9868 | 1.0000 |
| factor(X23.CS2)=0 | 5.81 | 196 | 0 | 2 | 0.9955 | 0.9868 | 1.0000 |
| factor(X23.CS2)=0 | 5.84 | 194 | 0 | 2 | 0.9955 | 0.9868 | 1.0000 |
| factor(X23.CS2)=0 | 5.85 | 192 | 0 | 1 | 0.9955 | 0.9868 | 1.0000 |
| factor(X23.CS2)=0 | 5.88 | 191 | 0 | 1 | 0.9955 | 0.9868 | 1.0000 |
| factor(X23.CS2)=0 | 5.89 | 190 | 0 | 1 | 0.9955 | 0.9868 | 1.0000 |
| factor(X23.CS2)=0 | 5.92 | 189 | 0 | 1 | 0.9955 | 0.9868 | 1.0000 |
| factor(X23.CS2)=0 | 5.93 | 188 | 0 | 1 | 0.9955 | 0.9868 | 1.0000 |
| factor(X23.CS2)=0 | 5.94 | 187 | 0 | 2 | 0.9955 | 0.9868 | 1.0000 |
| factor(X23.CS2)=0 | 5.95 | 185 | 0 | 3 | 0.9955 | 0.9868 | 1.0000 |
| factor(X23.CS2)=0 | 5.96 | 182 | 0 | 1 | 0.9955 | 0.9868 | 1.0000 |
| factor(X23.CS2)=0 | 5.98 | 181 | 0 | 1 | 0.9955 | 0.9868 | 1.0000 |
| factor(X23.CS2)=0 | 6 | 180 | 0 | 6 | 0.9955 | 0.9868 | 1.0000 |
| factor(X23.CS2)=0 | 6.01 | 174 | 0 | 1 | 0.9955 | 0.9868 | 1.0000 |
| factor(X23.CS2)=0 | 6.02 | 173 | 0 | 1 | 0.9955 | 0.9868 | 1.0000 |
| factor(X23.CS2)=0 | 6.04 | 172 | 0 | 1 | 0.9955 | 0.9868 | 1.0000 |
| factor(X23.CS2)=0 | 6.07 | 171 | 0 | 1 | 0.9955 | 0.9868 | 1.0000 |
| factor(X23.CS2)=0 | 6.08 | 170 | 0 | 1 | 0.9955 | 0.9868 | 1.0000 |
| factor(X23.CS2)=0 | 6.12 | 169 | 0 | 2 | 0.9955 | 0.9868 | 1.0000 |
| factor(X23.CS2)=0 | 6.15 | 167 | 0 | 3 | 0.9955 | 0.9868 | 1.0000 |
| factor(X23.CS2)=0 | 6.16 | 164 | 0 | 1 | 0.9955 | 0.9868 | 1.0000 |
| factor(X23.CS2)=0 | 6.17 | 163 | 0 | 1 | 0.9955 | 0.9868 | 1.0000 |
| factor(X23.CS2)=0 | 6.18 | 162 | 0 | 2 | 0.9955 | 0.9868 | 1.0000 |
| factor(X23.CS2)=0 | 6.22 | 160 | 0 | 1 | 0.9955 | 0.9868 | 1.0000 |
| factor(X23.CS2)=0 | 6.25 | 159 | 0 | 2 | 0.9955 | 0.9868 | 1.0000 |
| factor(X23.CS2)=0 | 6.27 | 157 | 0 | 1 | 0.9955 | 0.9868 | 1.0000 |
| factor(X23.CS2)=0 | 6.28 | 156 | 0 | 1 | 0.9955 | 0.9868 | 1.0000 |
| factor(X23.CS2)=0 | 6.29 | 155 | 0 | 1 | 0.9955 | 0.9868 | 1.0000 |
| factor(X23.CS2)=0 | 6.32 | 154 | 0 | 1 | 0.9955 | 0.9868 | 1.0000 |
| factor(X23.CS2)=0 | 6.35 | 153 | 0 | 2 | 0.9955 | 0.9868 | 1.0000 |
| factor(X23.CS2)=0 | 6.36 | 151 | 0 | 1 | 0.9955 | 0.9868 | 1.0000 |
| factor(X23.CS2)=0 | 6.37 | 150 | 0 | 2 | 0.9955 | 0.9868 | 1.0000 |
| factor(X23.CS2)=0 | 6.38 | 148 | 0 | 1 | 0.9955 | 0.9868 | 1.0000 |
| factor(X23.CS2)=0 | 6.39 | 147 | 0 | 3 | 0.9955 | 0.9868 | 1.0000 |
| factor(X23.CS2)=0 | 6.4 | 144 | 0 | 2 | 0.9955 | 0.9868 | 1.0000 |
| factor(X23.CS2)=0 | 6.42 | 142 | 0 | 3 | 0.9955 | 0.9868 | 1.0000 |
| factor(X23.CS2)=0 | 6.45 | 139 | 3 | 1 | 0.9740 | 0.9488 | 0.9999 |
| factor(X23.CS2)=0 | 6.46 | 135 | 0 | 2 | 0.9740 | 0.9488 | 0.9999 |
| factor(X23.CS2)=0 | 6.47 | 133 | 0 | 3 | 0.9740 | 0.9488 | 0.9999 |
| factor(X23.CS2)=0 | 6.48 | 130 | 0 | 2 | 0.9740 | 0.9488 | 0.9999 |
| factor(X23.CS2)=0 | 6.49 | 128 | 0 | 1 | 0.9740 | 0.9488 | 0.9999 |
| factor(X23.CS2)=0 | 6.5 | 127 | 0 | 2 | 0.9740 | 0.9488 | 0.9999 |
| factor(X23.CS2)=0 | 6.51 | 125 | 0 | 2 | 0.9740 | 0.9488 | 0.9999 |
| factor(X23.CS2)=0 | 6.52 | 123 | 0 | 1 | 0.9740 | 0.9488 | 0.9999 |
| factor(X23.CS2)=0 | 6.53 | 122 | 0 | 1 | 0.9740 | 0.9488 | 0.9999 |
| factor(X23.CS2)=0 | 6.54 | 121 | 0 | 1 | 0.9740 | 0.9488 | 0.9999 |
| factor(X23.CS2)=0 | 6.55 | 120 | 0 | 1 | 0.9740 | 0.9488 | 0.9999 |
| factor(X23.CS2)=0 | 6.57 | 119 | 0 | 2 | 0.9740 | 0.9488 | 0.9999 |
| factor(X23.CS2)=0 | 6.58 | 117 | 0 | 2 | 0.9740 | 0.9488 | 0.9999 |
| factor(X23.CS2)=0 | 6.59 | 115 | 0 | 1 | 0.9740 | 0.9488 | 0.9999 |
| factor(X23.CS2)=0 | 6.6 | 114 | 0 | 1 | 0.9740 | 0.9488 | 0.9999 |
| factor(X23.CS2)=0 | 6.63 | 113 | 0 | 1 | 0.9740 | 0.9488 | 0.9999 |
| factor(X23.CS2)=0 | 6.64 | 112 | 0 | 2 | 0.9740 | 0.9488 | 0.9999 |
| factor(X23.CS2)=0 | 6.65 | 110 | 0 | 3 | 0.9740 | 0.9488 | 0.9999 |
| factor(X23.CS2)=0 | 6.68 | 107 | 0 | 1 | 0.9740 | 0.9488 | 0.9999 |
| factor(X23.CS2)=0 | 6.69 | 106 | 0 | 1 | 0.9740 | 0.9488 | 0.9999 |
| factor(X23.CS2)=0 | 6.73 | 105 | 0 | 1 | 0.9740 | 0.9488 | 0.9999 |
| factor(X23.CS2)=0 | 6.75 | 104 | 0 | 3 | 0.9740 | 0.9488 | 0.9999 |
| factor(X23.CS2)=0 | 6.77 | 101 | 0 | 1 | 0.9740 | 0.9488 | 0.9999 |
| factor(X23.CS2)=0 | 6.78 | 100 | 0 | 1 | 0.9740 | 0.9488 | 0.9999 |
| factor(X23.CS2)=0 | 6.79 | 99 | 0 | 1 | 0.9740 | 0.9488 | 0.9999 |
| factor(X23.CS2)=0 | 6.81 | 98 | 0 | 2 | 0.9740 | 0.9488 | 0.9999 |
| factor(X23.CS2)=0 | 6.85 | 96 | 0 | 1 | 0.9740 | 0.9488 | 0.9999 |
| factor(X23.CS2)=0 | 6.89 | 95 | 0 | 2 | 0.9740 | 0.9488 | 0.9999 |
| factor(X23.CS2)=0 | 6.91 | 93 | 0 | 1 | 0.9740 | 0.9488 | 0.9999 |
| factor(X23.CS2)=0 | 6.92 | 92 | 0 | 1 | 0.9740 | 0.9488 | 0.9999 |
| factor(X23.CS2)=0 | 6.94 | 91 | 0 | 1 | 0.9740 | 0.9488 | 0.9999 |
| factor(X23.CS2)=0 | 6.96 | 90 | 0 | 4 | 0.9740 | 0.9488 | 0.9999 |
| factor(X23.CS2)=0 | 6.98 | 86 | 0 | 1 | 0.9740 | 0.9488 | 0.9999 |
| factor(X23.CS2)=0 | 7 | 85 | 0 | 3 | 0.9740 | 0.9488 | 0.9999 |
| factor(X23.CS2)=0 | 7.08 | 82 | 0 | 1 | 0.9740 | 0.9488 | 0.9999 |
| factor(X23.CS2)=0 | 7.14 | 81 | 0 | 1 | 0.9740 | 0.9488 | 0.9999 |
| factor(X23.CS2)=0 | 7.16 | 80 | 0 | 1 | 0.9740 | 0.9488 | 0.9999 |
| factor(X23.CS2)=0 | 7.2 | 79 | 0 | 1 | 0.9740 | 0.9488 | 0.9999 |
| factor(X23.CS2)=0 | 7.21 | 78 | 0 | 1 | 0.9740 | 0.9488 | 0.9999 |
| factor(X23.CS2)=0 | 7.26 | 77 | 0 | 3 | 0.9740 | 0.9488 | 0.9999 |
| factor(X23.CS2)=0 | 7.29 | 74 | 0 | 1 | 0.9740 | 0.9488 | 0.9999 |
| factor(X23.CS2)=0 | 7.35 | 73 | 0 | 2 | 0.9740 | 0.9488 | 0.9999 |
| factor(X23.CS2)=0 | 7.36 | 71 | 0 | 4 | 0.9740 | 0.9488 | 0.9999 |
| factor(X23.CS2)=0 | 7.37 | 67 | 0 | 1 | 0.9740 | 0.9488 | 0.9999 |
| factor(X23.CS2)=0 | 7.38 | 66 | 0 | 1 | 0.9740 | 0.9488 | 0.9999 |
| factor(X23.CS2)=0 | 7.39 | 65 | 0 | 2 | 0.9740 | 0.9488 | 0.9999 |
| factor(X23.CS2)=0 | 7.4 | 63 | 0 | 1 | 0.9740 | 0.9488 | 0.9999 |
| factor(X23.CS2)=0 | 7.41 | 62 | 0 | 1 | 0.9740 | 0.9488 | 0.9999 |
| factor(X23.CS2)=0 | 7.42 | 61 | 0 | 1 | 0.9740 | 0.9488 | 0.9999 |
| factor(X23.CS2)=0 | 7.45 | 60 | 0 | 1 | 0.9740 | 0.9488 | 0.9999 |
| factor(X23.CS2)=0 | 7.47 | 59 | 0 | 1 | 0.9740 | 0.9488 | 0.9999 |
| factor(X23.CS2)=0 | 7.48 | 58 | 0 | 2 | 0.9740 | 0.9488 | 0.9999 |
| factor(X23.CS2)=0 | 7.5 | 56 | 0 | 1 | 0.9740 | 0.9488 | 0.9999 |
| factor(X23.CS2)=0 | 7.53 | 55 | 0 | 1 | 0.9740 | 0.9488 | 0.9999 |
| factor(X23.CS2)=0 | 7.54 | 54 | 0 | 1 | 0.9740 | 0.9488 | 0.9999 |
| factor(X23.CS2)=0 | 7.55 | 53 | 0 | 1 | 0.9740 | 0.9488 | 0.9999 |
| factor(X23.CS2)=0 | 7.56 | 52 | 0 | 2 | 0.9740 | 0.9488 | 0.9999 |
| factor(X23.CS2)=0 | 7.58 | 50 | 0 | 4 | 0.9740 | 0.9488 | 0.9999 |
| factor(X23.CS2)=0 | 7.59 | 46 | 0 | 1 | 0.9740 | 0.9488 | 0.9999 |
| factor(X23.CS2)=0 | 7.6 | 45 | 0 | 2 | 0.9740 | 0.9488 | 0.9999 |
| factor(X23.CS2)=0 | 7.61 | 43 | 0 | 1 | 0.9740 | 0.9488 | 0.9999 |
| factor(X23.CS2)=0 | 7.62 | 42 | 0 | 1 | 0.9740 | 0.9488 | 0.9999 |
| factor(X23.CS2)=0 | 7.67 | 41 | 0 | 1 | 0.9740 | 0.9488 | 0.9999 |
| factor(X23.CS2)=0 | 7.68 | 40 | 0 | 1 | 0.9740 | 0.9488 | 0.9999 |
| factor(X23.CS2)=0 | 7.69 | 39 | 0 | 2 | 0.9740 | 0.9488 | 0.9999 |
| factor(X23.CS2)=0 | 7.73 | 37 | 0 | 2 | 0.9740 | 0.9488 | 0.9999 |
| factor(X23.CS2)=0 | 7.74 | 35 | 0 | 3 | 0.9740 | 0.9488 | 0.9999 |
| factor(X23.CS2)=0 | 7.81 | 32 | 0 | 1 | 0.9740 | 0.9488 | 0.9999 |
| factor(X23.CS2)=0 | 7.82 | 31 | 0 | 1 | 0.9740 | 0.9488 | 0.9999 |
| factor(X23.CS2)=0 | 7.94 | 30 | 0 | 2 | 0.9740 | 0.9488 | 0.9999 |
| factor(X23.CS2)=0 | 7.98 | 28 | 0 | 1 | 0.9740 | 0.9488 | 0.9999 |
| factor(X23.CS2)=0 | 7.99 | 27 | 1 | 1 | 0.9380 | 0.8672 | 1.0000 |
| factor(X23.CS2)=0 | 8.01 | 25 | 0 | 1 | 0.9380 | 0.8672 | 1.0000 |
| factor(X23.CS2)=0 | 8.07 | 24 | 0 | 1 | 0.9380 | 0.8672 | 1.0000 |
| factor(X23.CS2)=0 | 8.15 | 23 | 0 | 1 | 0.9380 | 0.8672 | 1.0000 |
| factor(X23.CS2)=0 | 8.17 | 22 | 0 | 1 | 0.9380 | 0.8672 | 1.0000 |
| factor(X23.CS2)=0 | 8.21 | 21 | 0 | 1 | 0.9380 | 0.8672 | 1.0000 |
| factor(X23.CS2)=0 | 8.26 | 20 | 0 | 1 | 0.9380 | 0.8672 | 1.0000 |
| factor(X23.CS2)=0 | 8.27 | 19 | 0 | 1 | 0.9380 | 0.8672 | 1.0000 |
| factor(X23.CS2)=0 | 8.28 | 18 | 0 | 1 | 0.9380 | 0.8672 | 1.0000 |
| factor(X23.CS2)=0 | 8.29 | 17 | 0 | 1 | 0.9380 | 0.8672 | 1.0000 |
| factor(X23.CS2)=0 | 8.48 | 16 | 0 | 1 | 0.9380 | 0.8672 | 1.0000 |
| factor(X23.CS2)=0 | 8.49 | 15 | 0 | 2 | 0.9380 | 0.8672 | 1.0000 |
| factor(X23.CS2)=0 | 8.5 | 13 | 0 | 1 | 0.9380 | 0.8672 | 1.0000 |
| factor(X23.CS2)=0 | 8.53 | 12 | 0 | 1 | 0.9380 | 0.8672 | 1.0000 |
| factor(X23.CS2)=0 | 8.72 | 11 | 0 | 1 | 0.9380 | 0.8672 | 1.0000 |
| factor(X23.CS2)=0 | 8.84 | 10 | 0 | 1 | 0.9380 | 0.8672 | 1.0000 |
| factor(X23.CS2)=0 | 8.88 | 9 | 0 | 2 | 0.9380 | 0.8672 | 1.0000 |
| factor(X23.CS2)=0 | 9.04 | 7 | 0 | 1 | 0.9380 | 0.8672 | 1.0000 |
| factor(X23.CS2)=0 | 9.06 | 6 | 0 | 2 | 0.9380 | 0.8672 | 1.0000 |
| factor(X23.CS2)=0 | 9.08 | 4 | 0 | 1 | 0.9380 | 0.8672 | 1.0000 |
| factor(X23.CS2)=0 | 9.26 | 3 | 0 | 1 | 0.9380 | 0.8672 | 1.0000 |
| factor(X23.CS2)=0 | 9.61 | 2 | 0 | 1 | 0.9380 | 0.8672 | 1.0000 |
| factor(X23.CS2)=0 | 9.95 | 1 | 0 | 1 | 0.9380 | 0.8672 | 1.0000 |
| factor(X23.CS2)=1 | 6.56 | 1 | 1 | 0 | 0.0000 |  |  |
| factor(X23.CS2)=2 | 5.12 | 39 | 1 | 0 | 0.9744 | 0.9260 | 1.0000 |
| factor(X23.CS2)=2 | 5.2 | 38 | 0 | 1 | 0.9744 | 0.9260 | 1.0000 |
| factor(X23.CS2)=2 | 5.26 | 37 | 0 | 1 | 0.9744 | 0.9260 | 1.0000 |
| factor(X23.CS2)=2 | 5.33 | 36 | 0 | 1 | 0.9744 | 0.9260 | 1.0000 |
| factor(X23.CS2)=2 | 5.34 | 35 | 0 | 1 | 0.9744 | 0.9260 | 1.0000 |
| factor(X23.CS2)=2 | 5.36 | 34 | 0 | 1 | 0.9744 | 0.9260 | 1.0000 |
| factor(X23.CS2)=2 | 5.38 | 33 | 0 | 1 | 0.9744 | 0.9260 | 1.0000 |
| factor(X23.CS2)=2 | 5.4 | 32 | 0 | 1 | 0.9744 | 0.9260 | 1.0000 |
| factor(X23.CS2)=2 | 5.42 | 31 | 0 | 1 | 0.9744 | 0.9260 | 1.0000 |
| factor(X23.CS2)=2 | 5.48 | 30 | 0 | 1 | 0.9744 | 0.9260 | 1.0000 |
| factor(X23.CS2)=2 | 5.55 | 29 | 0 | 1 | 0.9744 | 0.9260 | 1.0000 |
| factor(X23.CS2)=2 | 5.81 | 28 | 1 | 0 | 0.9396 | 0.8608 | 1.0000 |
| factor(X23.CS2)=2 | 5.89 | 27 | 0 | 1 | 0.9396 | 0.8608 | 1.0000 |
| factor(X23.CS2)=2 | 6.04 | 26 | 0 | 1 | 0.9396 | 0.8608 | 1.0000 |
| factor(X23.CS2)=2 | 6.16 | 25 | 0 | 1 | 0.9396 | 0.8608 | 1.0000 |
| factor(X23.CS2)=2 | 6.19 | 24 | 0 | 1 | 0.9396 | 0.8608 | 1.0000 |
| factor(X23.CS2)=2 | 6.2 | 23 | 0 | 1 | 0.9396 | 0.8608 | 1.0000 |
| factor(X23.CS2)=2 | 6.38 | 22 | 0 | 1 | 0.9396 | 0.8608 | 1.0000 |
| factor(X23.CS2)=2 | 6.39 | 21 | 0 | 1 | 0.9396 | 0.8608 | 1.0000 |
| factor(X23.CS2)=2 | 6.4 | 20 | 0 | 1 | 0.9396 | 0.8608 | 1.0000 |
| factor(X23.CS2)=2 | 6.51 | 19 | 0 | 1 | 0.9396 | 0.8608 | 1.0000 |
| factor(X23.CS2)=2 | 6.56 | 18 | 0 | 1 | 0.9396 | 0.8608 | 1.0000 |
| factor(X23.CS2)=2 | 6.79 | 17 | 0 | 1 | 0.9396 | 0.8608 | 1.0000 |
| factor(X23.CS2)=2 | 6.87 | 16 | 0 | 1 | 0.9396 | 0.8608 | 1.0000 |
| factor(X23.CS2)=2 | 7.12 | 15 | 0 | 1 | 0.9396 | 0.8608 | 1.0000 |
| factor(X23.CS2)=2 | 7.27 | 14 | 0 | 1 | 0.9396 | 0.8608 | 1.0000 |
| factor(X23.CS2)=2 | 7.36 | 13 | 0 | 2 | 0.9396 | 0.8608 | 1.0000 |
| factor(X23.CS2)=2 | 7.46 | 11 | 0 | 1 | 0.9396 | 0.8608 | 1.0000 |
| factor(X23.CS2)=2 | 7.48 | 10 | 0 | 1 | 0.9396 | 0.8608 | 1.0000 |
| factor(X23.CS2)=2 | 7.72 | 9 | 0 | 1 | 0.9396 | 0.8608 | 1.0000 |
| factor(X23.CS2)=2 | 7.74 | 8 | 0 | 2 | 0.9396 | 0.8608 | 1.0000 |
| factor(X23.CS2)=2 | 7.9 | 6 | 0 | 1 | 0.9396 | 0.8608 | 1.0000 |
| factor(X23.CS2)=2 | 8.06 | 5 | 0 | 1 | 0.9396 | 0.8608 | 1.0000 |
| factor(X23.CS2)=2 | 8.1 | 4 | 0 | 1 | 0.9396 | 0.8608 | 1.0000 |
| factor(X23.CS2)=2 | 8.36 | 3 | 0 | 1 | 0.9396 | 0.8608 | 1.0000 |
| factor(X23.CS2)=2 | 8.69 | 2 | 0 | 1 | 0.9396 | 0.8608 | 1.0000 |
| factor(X23.CS2)=2 | 8.88 | 1 | 0 | 1 | 0.9396 | 0.8608 | 1.0000 |

|  |  |  |  |  |  |  |  |  |  |
| --- | --- | --- | --- | --- | --- | --- | --- | --- | --- |
| X23.CS2 | records | n.max | n.start | events | \*rmean | \*se(rmean) | median | 0.95LCL | 0.95UCL |
| factor(X23.CS2)=0 | 297 | 297 | 297 | 5 | 8.781 | 0.045 | NA | NA | NA |
| factor(X23.CS2)=1 | 1 | 1 | 1 | 1 | 6.56 | 0 | 6.56 | NA | NA |
| factor(X23.CS2)=2 | 39 | 39 | 39 | 2 | 8.677 | 0.14 | NA | NA | NA |

Created by EmpowerStats (www.empowerstats.com) and R on 2026-03-21
